# Crop steering through osmotic stress can reduce height but reduced yield in medical *Cannabis*

**DOI:** 10.1186/s42238-025-00351-2

**Published:** 2025-11-18

**Authors:** Justin Allred, Brendan Fatzinger, Bruce Bugbee

**Affiliations:** Crop Physiology Laboratory, Utah State University, Logan, UT 84322-4820 USA

**Keywords:** Abiotic stress, Flower yield, Cannabinoids, Salinity tolerance, Harvest index

## Abstract

**Background:**

Precision water stress, achieved via osmotic stress, has the potential to control plant size and improve crop quality without reducing yield, but results across species and cultivars have been inconsistent.

**Methods:**

This study examined the effects of elevated salinity on two diverse *Cannabis sativa* cultivars, Trump and Cherry. One group (control group) was grown at 4 mS cm⁻¹ (-0.14 MPa), one group at 8 mS cm⁻¹ (-0.28 MPa), and a third (hybrid) group at 8 mS cm⁻¹ (to reduce vegetative growth) until flowering and then reduced to 4 mS cm⁻¹ to minimize effect on flower yield.

**Results:**

Plant height was reduced 15% in both the high and hybrid treatments. Flower yield decreased by 20% in cv. ‘Trump’ in the hybrid treatment, but the decrease in yield in the high salinity treatment was not statistically significant. In cv. ‘Cherry’, flower yield declined by approximately 20% in both the constant high salinity and hybrid treatments. There was no difference in cannabinoid concentrations among treatments in either cultivar.

**Conclusions:**

These findings indicate that *Cannabis sativa* is highly tolerant to osmotic stress, but the response varies by cultivar. It is difficult to reduce plant size without reducing yield. There is no evidence that increased salinity altered cannabinoid concentration.

## Introduction

Precision water stress is a holy grail of crop production because it can reduce plant size and increase quality. In a classic study, Boyer (1970) reported the potential of mild water stress to reduce leaf expansion without reducing photosynthesis in soybeans, sunflowers, and corn. Multiple studies over the past 50 years have reported mixed results when trying to replicate this study (Berni et al. 2024).

Studies of precision water stress (often called “crop steering”) in *Cannabis* are limited but have been well studied in tomatoes. The results have been mixed. Wu and Kubota (2008) found that some tomato cultivars exhibited an increase in photosynthesis under moderate salinity (4.8 mS cm⁻¹), while higher salinity (8.4 mS cm⁻¹) reduced photosynthesis. Schwarz et al. (2002) reported reductions in both dry mass and photosynthesis as salinity increased. Romero-Aranda et al. (2000) reported a decrease in growth as salinity increased. Quality improvements under salinity stress have also been reported: increased sugar content (Agius et al. 2022), improved fruit flavor (Dorai et al. 2001; Chretien et al. 2000; Wu et al. 2004), with only slightly reduced yield. However, some studies noted reductions in fruit size (Dorai et al. 2001) or fruit weight, as seen in strawberries (Awang et al., 1995) and eggplant (Savvas et al., 1999) under elevated salinity conditions.

While drought and salinity are distinct abiotic plant stressors, both reduce water availability by lowering water potential. Drought can occur either through matric potential or salinity through osmotic potential. As such, salinity can be used to create precise levels of osmotic stress. Salinity is often associated with sodium chloride salt (NaCl), but multiple types of salts and nutrients can be used. Salinity is typically measured using electrical conductivity (EC), which is a measure of dissolved ions in solution (Richards 1954; Langenfeld et al. 2022).

Yield responses of *Cannabis* to salinity have been limited and variable. Caplan et al. (2019) reported increased yield under elevated salinity, while Yep et al. (2020) observed yield reductions in hydroponic culture. Gill et al. (2022) found increased levels of proline, a known osmoprotectant, under drought conditions and noted that *Cannabis* can survive at extremely low soil moisture levels. Growth and yield responses can be variable among genetic lines. *Cannabis* has a wide genetic diversity that is poorly characterized. Both osmotic and hormonal changes can both contribute to yield responses to crop steering. The high commercial value of *Cannabis* has led to growers to use osmotic stress to increase cannabinoid content (Maravaneh et al., 2022). Some studies have noted increases in cannabinoid content under moderate water stress (Caplan et al. 2019), whereas others have reported no effect or reductions under severe water stress (Morgan et al. 2024; Yep et al. 2020; Duong et al. 2023; Yuan et al. 2024). These findings suggest a threshold, where moderate stress may enhance secondary metabolite production, but exceeding a threshold could diminish both biomass and cannabinoid content (Rezghiyan et al. 2024; Morgan et al. 2024). Similar patterns have been reported in other high-value crops. Peppermint and lemon verbena both showed reduced essential oil content under drought conditions, largely due to reduced plant size (Tabatabaie et al., 2007).

Our objective was to quantify the effects of osmotic stress on *Cannabis* height, yield, harvest index and cannabinoid concentration in two diverse *Cannabis* cultivars.

## Materials and methods

### Propagation

**Fig. 1:**
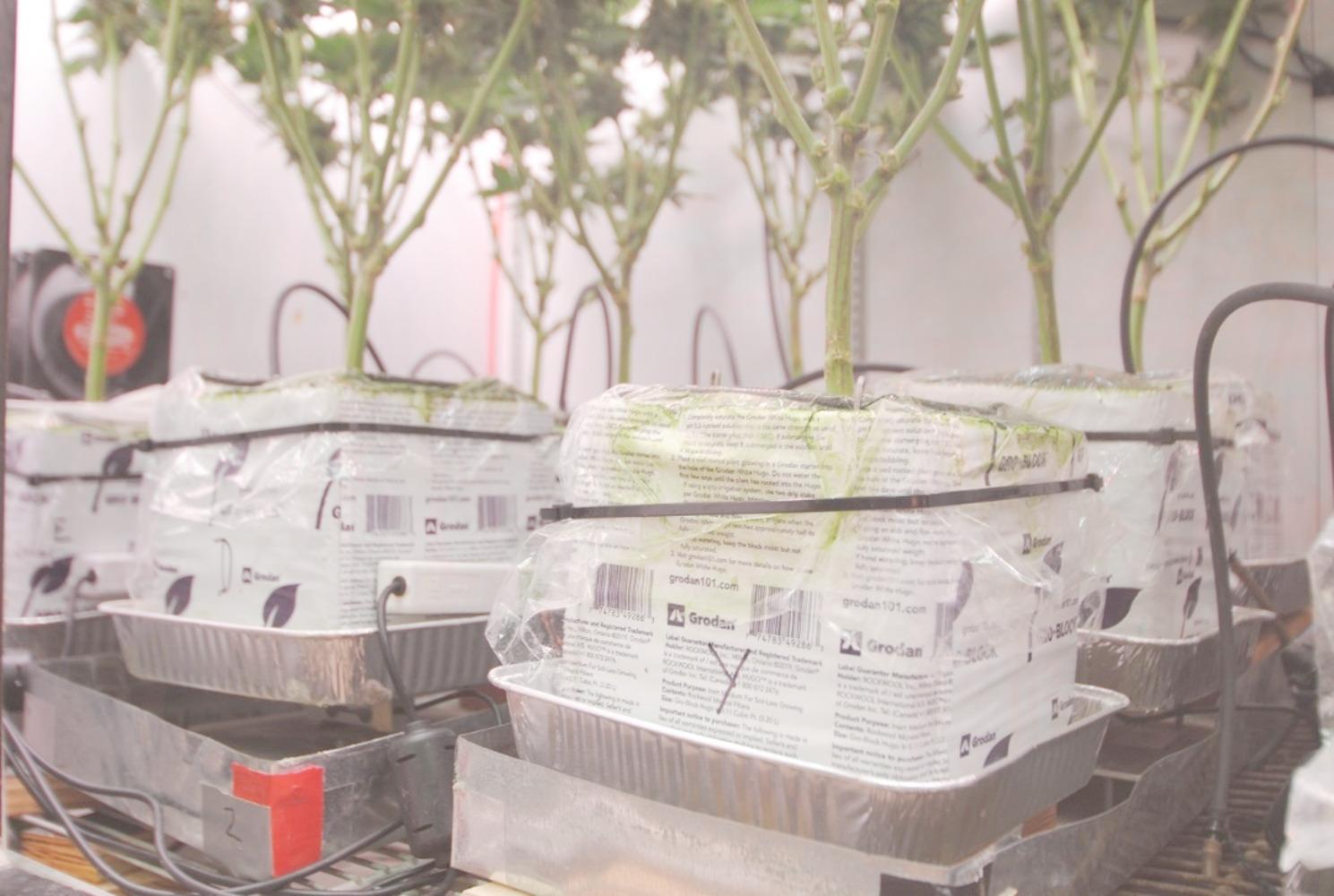
Cannabis ‘Trump’ growing in stone wool on load cells. Plastic wrap covered the tops of stone wool to minimize evaporation

**Fig. 2:**
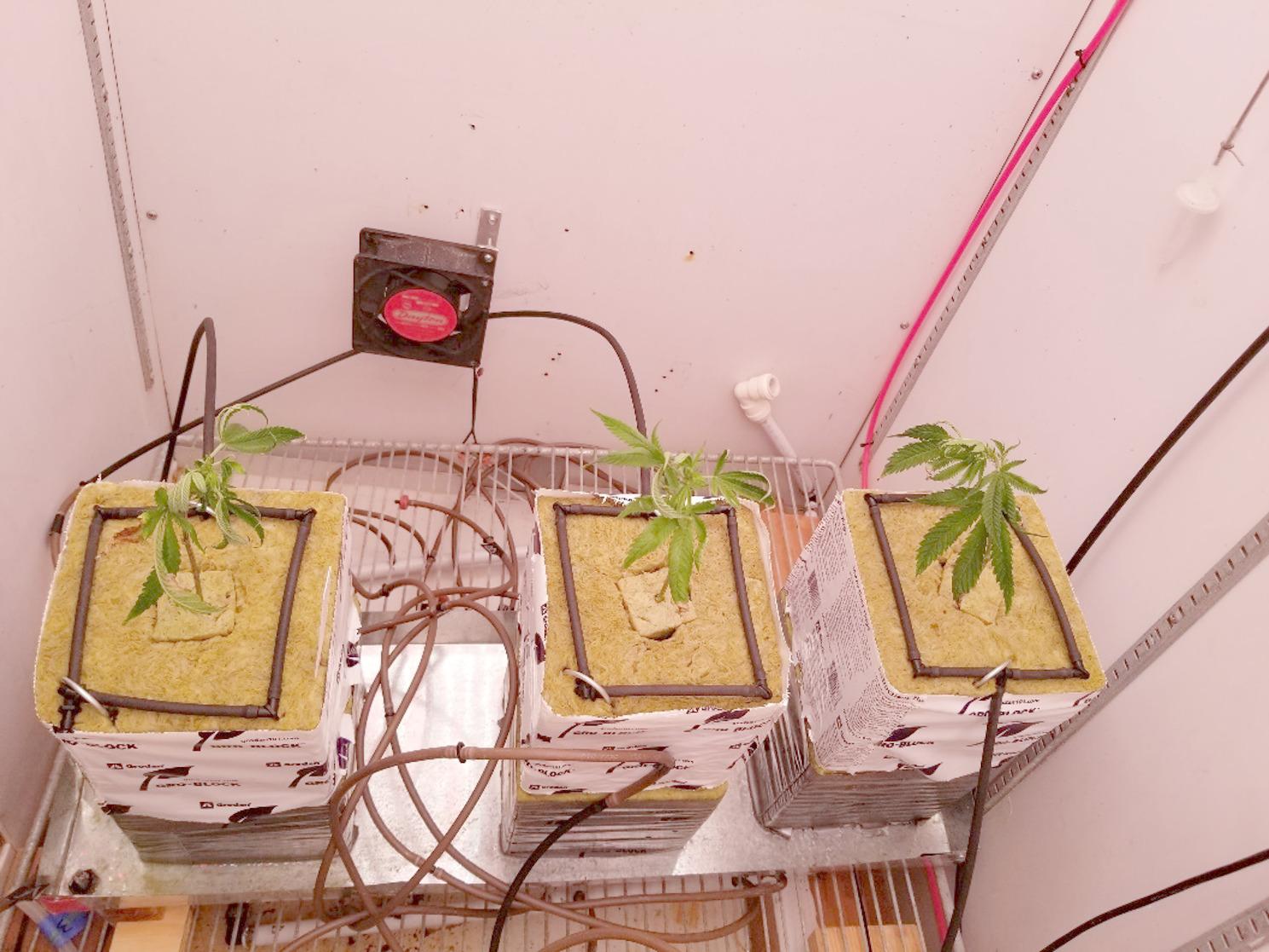
Top view of *Cannabis* ‘Trump’ growing in stone wool. Black tubing (three 0.5 mm-holes per side) was used to uniformly irrigate the stone wool blocks

Cuttings were propagated in 2 × 2 cm stone wool blocks (Grodan; Milton, ON). After 4 weeks of root development, they were placed into larger stone wool blocks (15 × 15 cm) and pruned to five nodes (Figs. 1 and 2). Two medical *Cannabis* cultivars (‘Trump’ and ‘Cherry’) were selected based on their commercial importance. In addition, cv. ‘Trump’ has thicker rigid stems and ‘Cherry’ has thinner, less rigid stems.

### Growth conditions and experimental design

Plants were grown in a growth chamber for one week at an 18-hour photoperiod before changing to an inductive 12-hour photoperiod for the eight weeks until harvest. The LED lighting was adjusted weekly by reducing the electrical current, (dimming) to maintain a constant PPFD (photosynthetic photon flux density) at 1000 µmol m^− 2^ s^− 1^. The spectral distribution was 0.1% UV, 10.8% Blue, 21.4% Green, 66.2% Red, 1.6% Far red. The temperature was 27 °C/22°C day/night and the vapor pressure deficit was 1 kPa. CO_2_ was elevated to 1200 ppm.

**Fig. 3:**
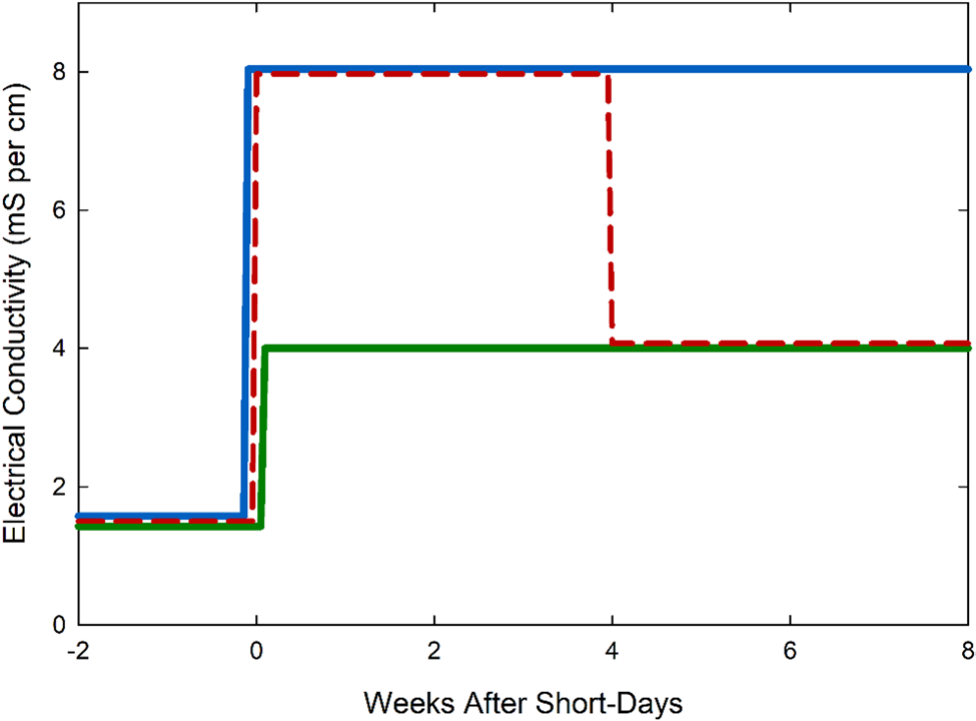
Timeline for the EC treatments. Blue is the high treatment (8 mS cm^−1^), green is the low treatment (4 mS cm^−1^), and red in the hybrid treatment (8 mS cm^−1^ for the first four weeks of short-days and 4 mS cm^−1^ for the last four weeks). All plants were started on nutrient solution with an EC of 1.5 mS cm^−1^ until rooted in the stone wool blocks

Plants were divided into three treatment groups with six replicate plants per group. The first group was watered with a constant nutrient solution concentration of 4 mS cm^− 1^, the second group was watered with 8 mS cm^− 1^, and the third (hybrid) group was watered with 8 mS cm^− 1^ solution for the first four weeks of short-days and then watered with 4 mS cm^− 1^ for the remaining four weeks (Fig. 3). This hybrid treatment was selected because stem elongation occurrs during the first four weeks of growth. The goal was to reduce plant height without reducing yield. Tables 1 and 2 show the concentration of the nutrient solutions.

**Table 1:** Fertilizer salts and molar concentrations used to make the nutrient solutions. K_2_SiO_3_ and micronutrients were the same in both treatment solutions

Macro	4 mS cm^− 1^ (mM)	8 mS cm^− 1^ (mM)
*Ca(NO*_*3*_*)*_*2*_	9	18
*KNO*_*3*_	12	24
*KH*_*2*_*PO*_*4*_	3	6
*MgSO*_*4*_	2.4	4.8
*K*_*2*_*SiO*_*3*_	0.6	0.6
*HNO*_*3*_	3	6

Micro	4 mS cm^− 1^ (µM)	8 mS cm^− 1^ (µM)
*Fe-DTPA*	7	7
*Mn-EDTA*	3	3
*ZnCl*_*2*_	3	3
*H*_*3*_*BO*_*3*_	40	40
*Cu-EDTA*	4	4
*Na*_*2*_*MoO*_*4*_	0.1	0.1
*NiCl*_*2*_	0.1	0.1

**Table 2:** Elemental composition of nutrient solutions

Macro	4 mS cm^− 1^ (ppm)	8 mS cm^− 1^ (ppm)
*N*	462	925
*P*	93	186
*K*	633	1220
*Ca*	361	721
*Mg*	58	117
*S*	77	154
*Si*	17	17

**Micro**	**4 mS cm**^**− 1**^ **(ppb)**	**8 mS cm**^**− 1**^ **(ppb)**
*Fe*	391	391
*Mn*	165	165
*Zn*	196	196
*B*	432	432
*Cu*	254	254
*Mo*	10	10
*Ni*	6	6
*Cl*	220	220
*Na*	5	5

### Osmotic stress and water potential

Osmotic potential (Ψ_s_) was calculated for the nutrient solution concentrations using the following equation (Richards 1954; Langenfeld et al. 2022):

$$\:EC\:\times\:\:-0.036={\Psi\:}s$$

where EC is expressed in mS cm^− 1^ and Ψ_s_ is in MPa. Osmotic potential values for the treatments were: −0.14 MPa for 4 mS cm^− 1^, −0.29 MPa for 8 mS cm^− 1^, and − 0.22 MPa for 8/4 mS cm^− 1^. The osmotic potential for the 8/4 mS cm^− 1^ treatment was calculated by taking the mean value for the 8-week growth period.

$$\begin{aligned}&\frac{\left(-0.14\:MPa\:\times\:4\:weeks\right)+\left(-0.29\:MPa\:\times\:4\:weeks\right)}{8\:weeks}\\&=\:-0.22\:MPa \end{aligned}$$

Based on van Genuchten (1980) model parameters reported by Chamindu et al. (2013), matric potential in stone wool at 50% VWC is approximately − 2.05 kPa (−0.002 MPa). Throughout the study, plants were maintained at or above field capacity (VWC > 50%), under which conditions matric potential (Ψm) was effectively near zero.

Irrigation was automated using electromagnetic sensors (model Teros 12, Meter Group; Pullman, WA) connected to a data acquisition system (model CR 1000x, Campbell Scientific, Logan UT). Due to the nature of stone wool blocks to turn hydrophobic if allowed to dry out after the start of irrigation, all blocks were kept above 50% volumetric water content (VWC). Irrigation frequency increased to several times an hour as the plant grew. Irrigation was more frequent in the day than in the night and larger plants used more water. VWC was monitored by moisture sensors, and VWC irrigation setpoints were established and periodically adjusted to ensure plants were not limited by water availability. Irrigation setpoints were used to turn irrigation on and off. For example, to control the setpoint at 55% VWC, irrigation would turn on for 1 s on, 15 s off, until VWC was above the 55% threshold, measured by the sensors. Each stone wool block had a sensor to ensure all plants did not have additional matric potential water stress (Fig. 1).

The stone wool blocks were placed on load cells (model LSP-5, Transducer Techniques; Temecula, CA) to provide a secondary way to monitor water content. Excess nutrient solution from the irrigation system drained free from the stone wool blocks, past the load cells, and into a collection tray. The leachate from three stone wool blocks (and subsequent plants), all from the same treatment, were collected in the same collection tray (*n* = 2).

Plants were inspected weekly for pests and were occasionally treated for spider mites (neem oil and Avid miticide) and powdery mildew (sulfur powder).

### Growth and harvest measurements

Plant height was measured and recorded three times a week from the top of the stone-wool block to the highest point on the plant. Leachate was measured for electrical conductivity (model HI98304, Hanna Instruments; Woonsocket RI) after 10 min of irrigation (about a liter of solution in all treatments).

Plants were harvested 8 weeks after the start of short days. Stems, leaves, and flowers were separated and dried at 80 °C for two days. For each group, subsamples (*n* = 2 for leaves and flowers for nutrient analysis; *n* = 2 for flower cannabinoid analysis) were randomly chosen for nutrient and cannabinoid content (Tables 2 and 3).

**Table 3:** Yield and quality for the three treatments for both *Cannabis* cultivars ‘Trump’ and ‘Cherry’. Sample size for dry flower, harvest index, and height were: *n* = 6 (‘Trump’) and *n* = 4 (‘Cherry’). Sample size for CBD, THC, and CBD/THC were *n* = 2 (both cultivars). Differences among cannabinoid concentrations were not statistically significant

	Treatment EC (mS cm^− 1^)	Dry Flower (g m^− 2^)	Harvest Index	Height (mm)	CBD (%)	THC (%)	CBD/THC
Trump	4	690^A^	0.56^A^	618^A^	11.6	0.39	30.5
	8/4	558^B^	0.59^B^	522^B^	11.1	0.39	28.8
	8	620^AB^	0.59^B^	544^C^	11.1	0.37	31.4
Cherry	4	706^a^	0.48^a^	498^a^	8.0	0.20	40.0
	8/4	560^b^	0.52^b^	440^b^	7.4	0.18	41.6
	8	557^b^	0.52^ab^	423^b^	8.5	0.23	38.8

### Statistical analysis

Statistical analyses were performed in R version 4.4.1 (R Core Team, 2024) within the RStudio environment (Posit Software, 2024). Data management and visualization were carried out using ‘tidyverse’ (Wickham et al. 2019). Analysis of variance (ANOVA) was conducted using the ‘car’ package (Fox & Weisberg, 2019), and Tukey’s Honest Significant Difference (HSD) tests for pairwise post-hoc comparisons were implemented via the ‘multcomp’ package (Hothorn et al. 2008).

## Results and discussion

Plant growth and development are influenced by multiple abiotic stresses, including temperature, drought, and salinity, which disrupt homeostasis, inhibit photosynthesis, and impair water and nutrient uptake. These stressors can act independently or interact to amplify one another’s effects, ultimately reducing plant performance and compromising crop yield and productivity (Zhang et al. 2022). By using a growth chamber, the effects of osmotic stress could be isolated from other environmental variables. The following results highlight how varying levels of osmotic stress influenced key agronomic traits, including plant height, flower yield, and crop quality.

### Effects on plant height

**Fig. 4:**
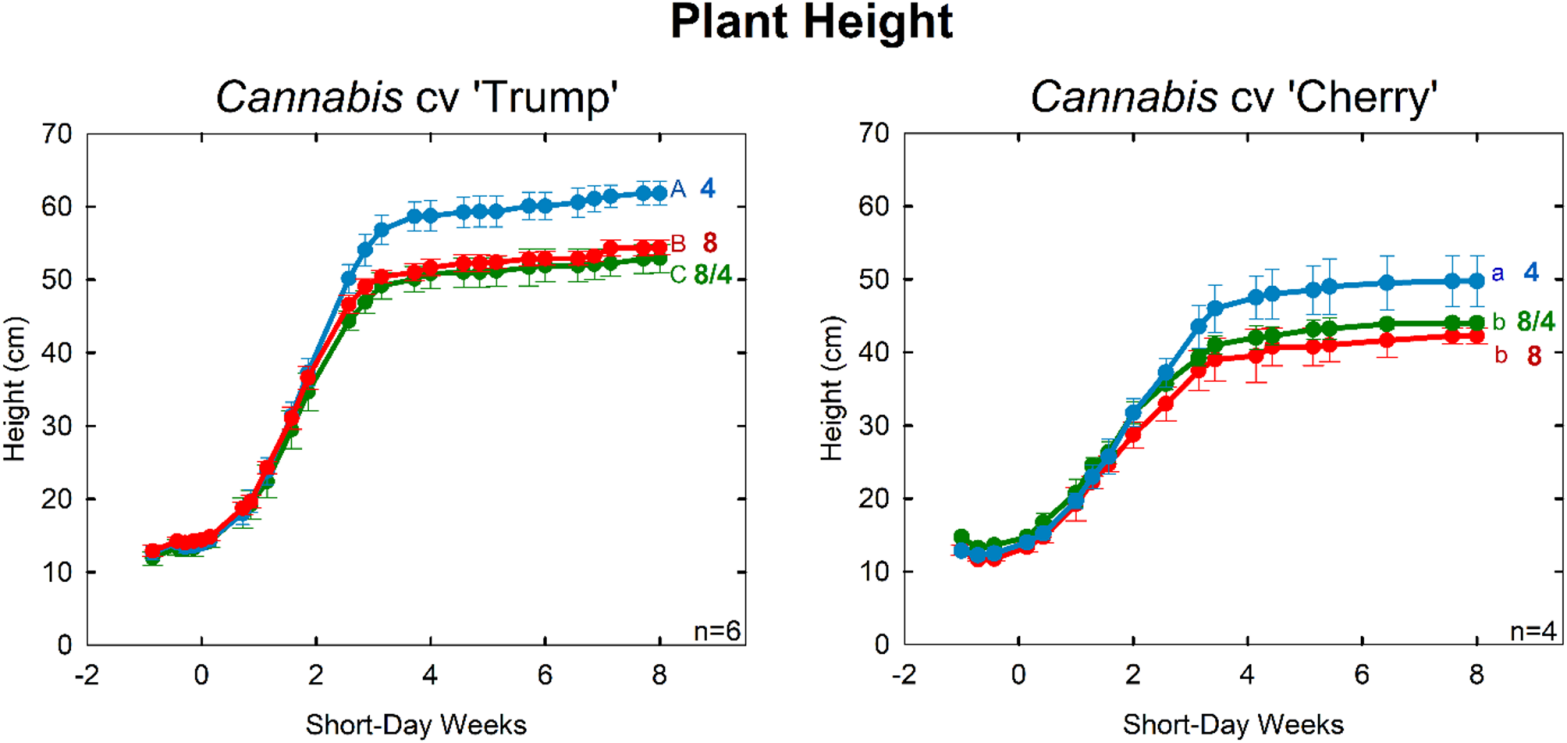
Plant height over time. There was a 15% reduction in plant height, in both cultivars, when treated with the high EC treatment. Cannabis ‘Trump’ on the left. Cannabis ‘Cherry’ on right. Error bars represent standard deviation (n = 6 for ‘Trump’; n = 4 for ‘Cherry’)

Plant height in the higher EC treatments was reduced by 15% in both cultivars (Table 3; Fig. 4). This height reduction demonstrates the potential of crop steering through osmotic stress. As expected, there was no significant height difference between the 8 mS cm^−1^ and 8/4 mS cm^−1^ treatment, indicating that osmotic stress during the flowering phase was not necessary for height reduction.

### Effect on yield and quality

**Fig. 5:**
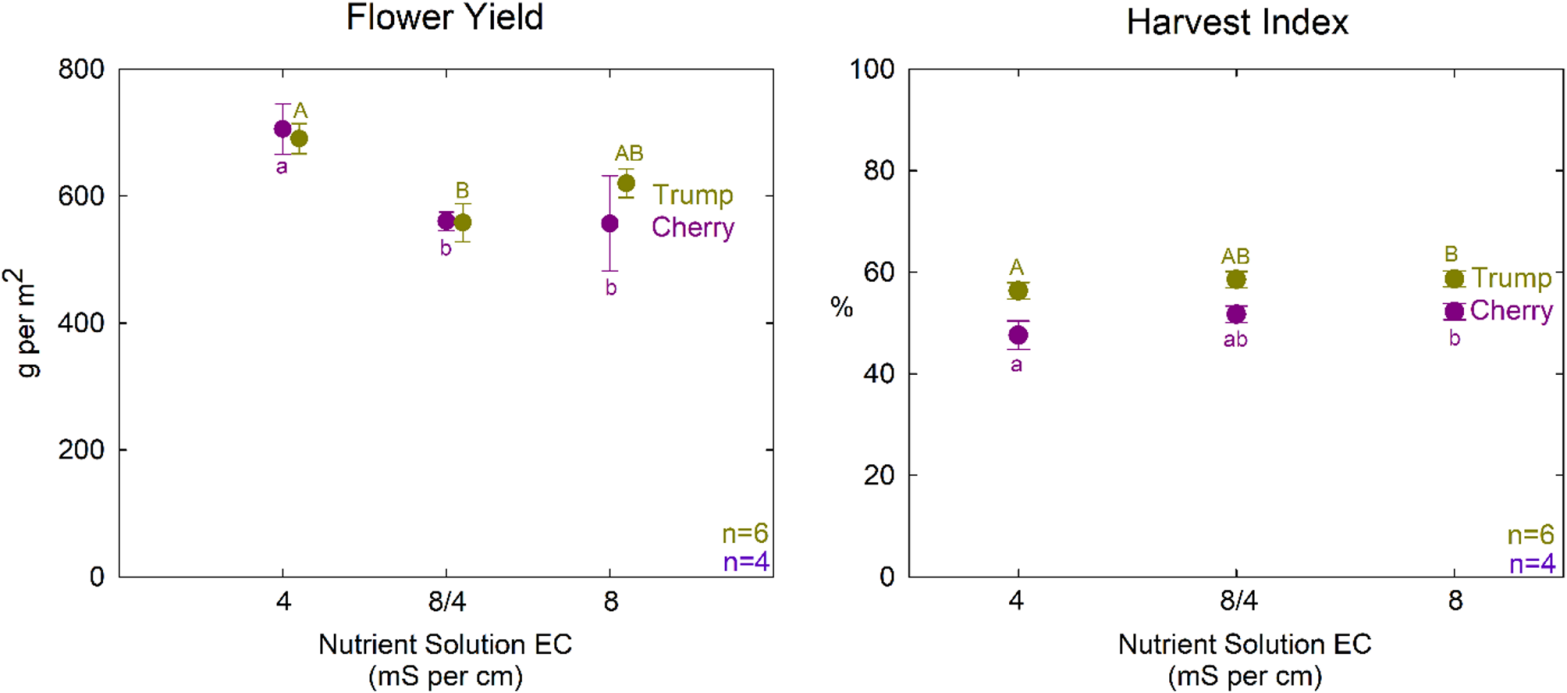
The effect of nutrient solution treatment on flower yield (left) and harvest index (right) for both cultivars. X-axis is the treatment electrical conductivity. Error bars represent standard deviation (n = 6 for ‘Trump’; n = 4 for ‘Cherry’)

The higher osmotic stress treatments (8/4 mS cm^−1^ and 8 mS cm^−1^) resulted in a 20% reduction in flower yield for ‘Cherry’. In contrast, in ‘Trump,’ only the 8/4 mS cm^−1^ treatment significantly reduced yield, while the 8 mS cm^−1^ treatment was not different from the other treatments (Table 3; Fig. 5). This suggests that ‘Trump’ may be more tolerant of osmotic stress. Plants respond to osmotic stress in multiple ways. The genetic differences could be the result of a differing ability for osmotic adjustment or altered carbon partitioning to roots. Root size and depth are important considerations in the field, but the small root zone in these studies suggests that root system variability did not contribute to the differences between these cultivars.

**Fig. 6:**
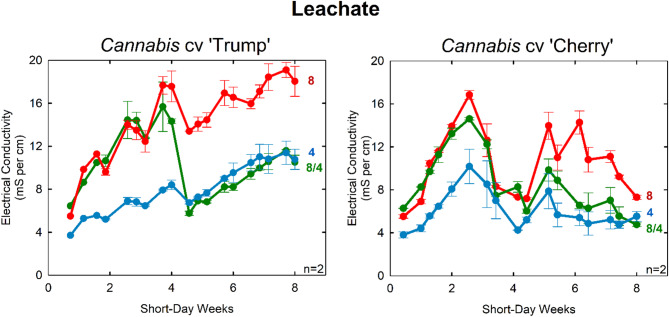
Leachate EC over time. The irrigation solution to the 8/4 treatment was changed after week four to reduce osmotic stress during flowering in both cultivars. Each stone wool block was leached three times a week with the respective nutrient solution. Leachate collected and measured. *Cannabis* ‘Trump’ on the left. *Cannabis* ‘Cherry’ on right. Error bars represent standard deviation (*n* = 2)

Notably, leachate collected from ‘Trump’ samples reached 20 mS cm⁻¹, corresponding to an osmotic stress of −0.72 MPa. This is nearly halfway to permanent wilting point (θ_wp_ = −1.5 MPa) (Fig. 6). This highlights the need for growers using osmotic stress for crop steering to maintain vigilance over irrigation management.

Interestingly, the harvest index in both cultivars was increased by the greater osmotic stress (Table 3; Fig. 5). This is due to the reduction in stem mass in the high EC treatments, despite a slight reduction in yield. This also suggests that osmotic stress during vegetative stages can steer biomass allocation toward flower production; a key consideration for optimizing yield in commercial *Cannabis* cultivation.

### Cannabinoid content

**Fig. 7:**
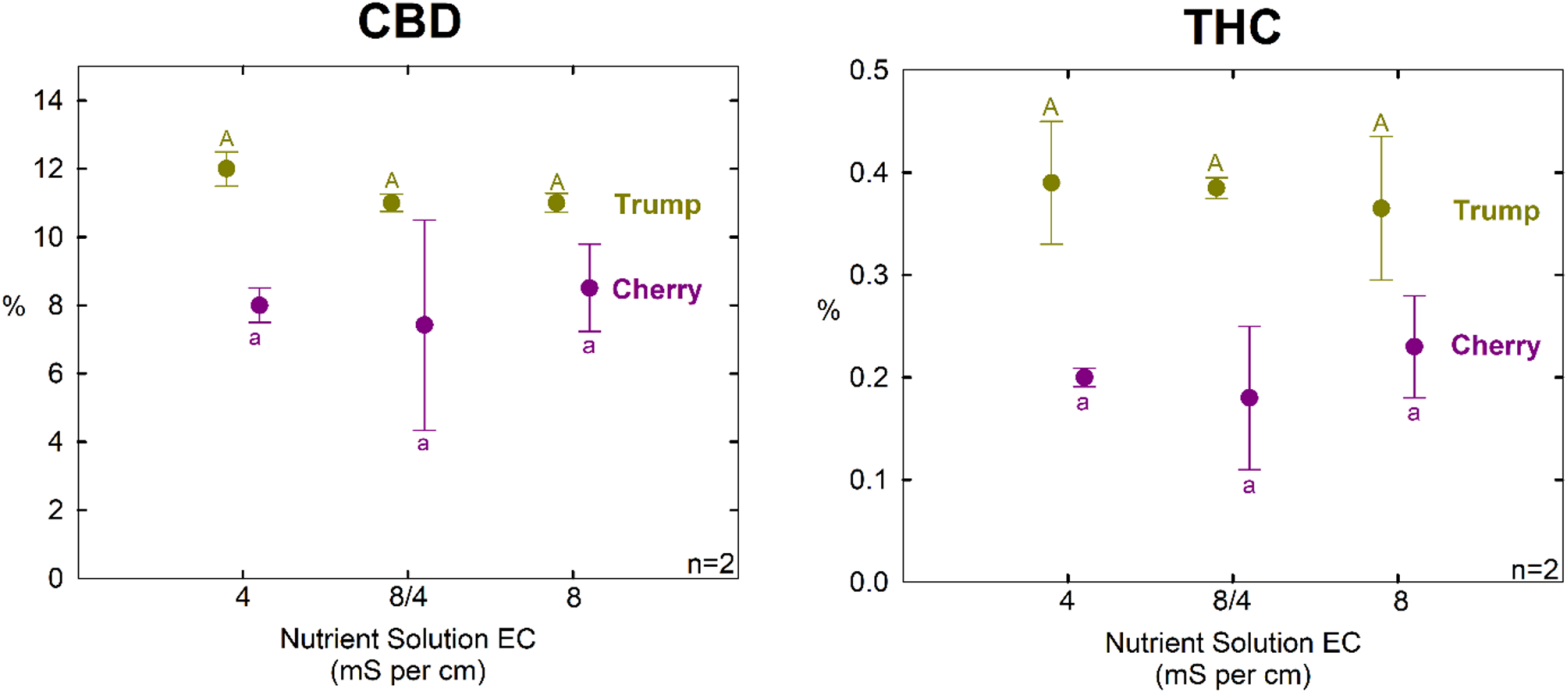
The effect of nutrient solution EC on CBD content (left) and THC content (right) for both cultivars. X-axis is the treatment EC solution used. Error bars represent the standard deviation (*n* = 2)

There was less than a 1% difference in CBD concentration, and less than a 0.1% difference in THC concentration among treatments. The ratio of CBD/THC was also not significantly different among treatments in either cultivar (Table 3; Fig. 7). No trends were apparent among treatments. The uniformity of the controlled environment facilitated low experimental error among replicate measurements. Although there were only two replicate per cultivar per treatment, the consistent results in each cultivar indicate that osmotic effects on cannabinoids are likely small.

These results indicate the challenge of using crop steering to increase product quality.

### Nutrient and media considerations

There were no statistically significant differences in leaf or flower nutrient concentrations among treatments and cultivars (Table 4). The small differences were not biologically important even had they been statistically significant. This indicates that the growth responses were the result of osmotic stress rather than nutrient availability or nutrient toxicity. These leaf tissue nutrient concentrations are within reported sufficiency ranges for *Cannabis* (Bryson and Mills 2015; Landis et al. 2019; Marschner 2012). These results are consistent with a comprehensive study by Herschowitz et al. (2025) who also found no difference leaf nutrient concentrations of *Cannabis* between an EC of 2 and 4 mS cm^−1^ in hydroponic culture. *Cannabis* appears to have the ability to regulate nutrient uptake across a wide range of nutrient concentrations in the root-zone. In contrast, Rahnama et al. (2010) found that growth of wheat was initially inhibited by salt-induced osmotic stress but was affected by the toxicity of sodium chloride.

Stone wool was used as the growing medium in this study due to its widespread use commercial *Cannabis* cultivation. This has been largely driven by its high water holding capacity and low cation exchange capacity (Bussell et al., 2004; Chamindu et al. 2013) which make it well-suited as a media for precise water stress and crop steering. (Table 4)

**Table 4:** Effect of electrical conductivity and cultivar on nutrient concentration in leaf and flower tissue (*n* = 2). There were no significant effects in either cultivar or treatments

Tissue	Cultivar	Treatment	N	P	K	Ca	Mg	S	Fe	Mn	Cu	Zn	B	Mo	Si
			**%**						**mg kg** ^− 1^						
*Flower*	*Trump*	4	3.7	0.8	3.7	2.0	0.5	0.9	137	122	14	80	31	3.5	380
		8/4	3.7	0.9	3.5	2.2	0.5	0.7	177	108	16	80	33	3.7	576
		8	3.7	0.8	3.3	1.9	0.4	0.7	118	121	15	86	33	3.8	464
	*Cherry*	4	4.8	1.3	4.0	1.9	0.5	0.5	154	77	15	65	34	1.9	556
		8/4	4.9	1.3	3.9	2.1	0.5	0.5	184	113	18	81	38	2.2	748
		8	4.9	1.3	4.3	2.0	0.5	0.5	164	125	18	91	36	1.9	791
*Leaf*	*Trump*	4	3.5	0.6	3.9	5.6	0.4	1.1	124	66	10	81	82	3.3	630
		8/4	3.4	0.6	3.5	6.1	0.5	1.2	82	56	11	78	93	2.9	641
		8	2.9	0.5	3.1	7.3	0.5	0.8	141	55	8	92	103	2.9	718
	*Cherry*	4	2.2	0.5	1.9	9.1	1.1	0.4	172	68	8	23	144	2.1	965
		8/4	2.7	0.6	2.9	8.6	0.8	0.3	183	87	8	38	118	1.3	810
		8	2.4	0.5	2.0	8.0	0.8	0.3	128	145	8	58	167	1.1	1293

## Conclusion

These findings confirm that *Cannabis sativa* is highly tolerant to osmotic stress and indicate that osmotic stress during vegetative growth can reduce plant height and increase harvest index. However, yield was reduced 20% in ‘Trump’ in the hybrid treatment and consistently reduced by 20% in ‘Cherry’. Collectively, these results indicate that ‘Trump’ is more tolerant to osmotic stress than ‘Cherry’, suggesting the potential for genetic selection for salinity tolerance in *Cannabis*. There was no effect on cannabinoid concentration in either cultivar.

Future research should also explore fine-tuning the timing of osmotic stress to mitigate yield loss while preserving the benefits of height control.

## Data Availability

No datasets were generated or analysed during the current study.

## Abbreviations

EC: Electrical conductivity
cv.: Cultivar
PPFD: Photosynthetic photon flux
LED: Light emitting diode
VWC: Volumetric water content
ppm: Parts per million
CBD: Cannabidiol
THC: Tetrahydrocannabinol
